# A Clinical Extensively-Drug Resistant (XDR) *Escherichia coli* and Role of Its β-Lactamase Genes

**DOI:** 10.3389/fmicb.2020.590357

**Published:** 2020-12-10

**Authors:** Mingyu Wang, Wenjia Wang, Yu Niu, Ting Liu, Ling Li, Mengge Zhang, Ziyun Li, Wenya Su, Fangyue Liu, Xuhua Zhang, Hai Xu

**Affiliations:** ^1^State Key Laboratory of Microbial Technology, Microbial Technology Institute, Shandong University, Qingdao, China; ^2^Laboratory Medicine Center, The Second Hospital of Shandong University, Jinan, China; ^3^Shandong Shian Chemical Co., Ltd., Dezhou, China

**Keywords:** antimicrobial resistance, extensively drug resistance, *Escherichia coli*, β-lactamase, β-lactamase inhibitor, multidrug resistant plasmid

## Abstract

An extensively-drug resistant (XDR) *Escherichia coli* W60 was isolated from the urine sample of a patient. The genetic basis for its XDR phenotype was investigated, particularly the basis for its resistance toward β-lactam/BLI (β-Lactamase Inhibitor) combinations. Following determination of the XDR phenotype, third generation genomic sequencing was performed to identify genetic structures in *E. coli* W60. Further cloning analysis was performed to identify determinants of β-lactam/BLI combination resistance. It was found that *E. coli* W60 is resistant to nearly all of the tested antibiotics including all commonly used β-lactam/BLI combinations. Analysis of the genomic structures in *E. coli* W60 showed two novel transferable plasmids are responsible for the resistance phenotypes. Further genetic analysis showed *bla*_NDM–5_ leads to high resistance to β-lactam/BLI combinations, which was enhanced by co-expressing *ble*_MBL_. pECW602 harbors a truncated *bla*_TEM_ that is not functional due to the loss of the N-terminal signal peptide coding region. Research performed in this work leads to several significant conclusions: the XDR phenotype of *E. coli* W60 can be attributed to the presence of transferable multidrug resistance plasmids; NDM-5 confers high resistance to β-lactam/BLI combinations; co-expression of *ble*_MBL_ enhances resistance caused by NDM-5; the signal peptides of TEM type β-lactamases are essential for their secretion and function. Findings of this work show the danger of transferable multidrug resistance plasmids and metallo-β-lactamases, both of which should be given more attention in the analysis and treatment of multidrug resistant pathogens.

## Introduction

*Escherichia coli* is one of the most common clinical bacteria, of which many isolates are pathogenic. *E. coli* can cause enteritis, urinary tract infection and many other diseases, leading to significant morbidity and mortality (Russo, 2003). In the past few decades, following the increased use of antibiotics, the resistance of clinical *E. coli* to antibiotics rises, making it difficult for treatment. In particular, many *E. coli* strains developed multi-, extensively- or pan-drug resistance (MDR, XDR, or PDR) phenotypes, posing a great challenge to infection treatment (Magiorakos et al., 2012; Du et al., 2017; Jeong et al., 2018; Lv et al., 2018). Therapeutic options to these antibiotic resistant *E. coli* strains include last-resort antibiotics such as carbapenems and tigecycline, along with those still under development (Karaiskos and Giamarellou, 2014).

β-lactam antibiotics are the most widely used antibiotics in the treatment of bacterial infection. However, antibiotic resistant bacteria often produce β-lactamase, inactivating β-lactams. To address this, β-lactamase inhibitors (BLI) were developed to reenable the use of β-lactam antibiotics. Today, the most commonly used BLIs include tazobactam, clavulanate, sulbactam, and avibactam (Ehmann et al., 2012). Effective β-lactam/BLI combinations include piperacillin–tazobactam, amoxicillin–clavulanate, ticarcillin-clavulanate, ampicillin–sulbactam, and ceftazidime–avibactam (Tooke et al., 2019). The use of these combinations has replaced other last-resort antibiotics to become the most popular option in treating β-lactam resistant bacteria infections.

Based on sequence homology, β-lactamases are divided into four classes A, B, C, and D (Ambler, 1980). Despite differing by their mechanisms, all β-lactamases deactivate β-lactams by hydrolytic opening of the β-lactam ring. TEM is one of the most prevalent and typical class A β-lactamases. It was discovered in as early as 1965 when a plasmid harboring *bla*_TEM–1_ was found (Datta and Kontomichalou, 1965). A large number of TEM variants have been identified to date that mediate resistance to most β-lactams (Paterson and Bonomo, 2005). Among β-lactamases, metallo-β-lactamases (MBL) such as New Delhi Metallo-β-Lactamases (NDMs) rank among the most detrimental for their ability to lead to resistance against not only β-lactams but also carbapenems, and unlike other serine β-lactamases that exploit a serine active site for hydrolysis, MBLs rely on zinc ions in their active site to facilitate hydrolytic reaction (Bebrone, 2007). This different mechanism of MBLs on β-lactam hydrolysis leads to the consensus that BLIs are ineffective against MBLs. However, experimental evidence for whether all common β-lactam/BLI combinations are ineffective against MBLs is still needed. Statistics in recent years show that the prevalence of NDMs is increasing worldwide (Bush, 2018). Since the discovery of NDM-1, a total of 24 different NDM variants have been identified, the coding genes of which (*bla*_NDM_) are hosted by a variety of bacteria, predominantly *Enterobacteriaceae* followed by other pathogenic bacteria such as *Acinetobacter spp*. (Wu et al., 2019). Transferable plasmids play an important role in the dissemination of *bla*_NDM_ by hosting and spreading of the gene through horizontal gene transfer (HGT) (Adamczuk et al., 2015; Wailan et al., 2015; Sugawara et al., 2017; Liu et al., 2019). This has led to the wide distribution of NDM worldwide, posing a severe threat to public health (Dortet et al., 2014; Dadashi et al., 2019; Wu et al., 2019).

In this study, an extensively-drug resistant (XDR) *E. coli* W60 was isolated from the urine sample of a patient following his bladder tumor surgery. This strain was found resistant to all tested antibiotics except tigecycline. In particular, *E. coli* W60 was found resistant to all commonly available β-lactam/BLI combinations. Whole-genome sequencing revealed that W60 hosts two novel transferable plasmids, the IncFIB-type plasmid pECW601 and the IncFII-type plasmid pECW602, and showed that the two multidrug resistance plasmids carry the main genetic determinants of antimicrobial resistance for *E. coli* W60. pECW601 contains the *bla*_NDM–5_ gene, which encodes the metallo-β-lactamase NDM-5. pECW602 contains a truncated *bla*_TEM_ gene. Further genetic analysis provides experimental evidence that NDM5 leads to resistance to β-lactam/BLI combinations and that the N-terminal 28 amino acids containing signal peptide appear essential for the functionality of TEM. This work provides a detailed insight into the resistance mechanisms of a clinical XDR *E. coli* strain, and provides evidence on the role of β-lactamase genes. In particular, this work demonstrates MBLs indeed renders BLIs ineffective, further stressing the danger of these now widespread β-lactamase genes.

## Materials and Methods

### Bacterial Strains

The strain *E. coli* W60 used in this study was isolated from a urine sample of a patient from the Second Hospital of Shandong University who had an infection after bladder tumor resection. The preliminary identification results of the hospital showed that the bacterium was resistant to multiple antibiotics, so further research was needed to develop a treatment plan for the patient. The handling and experiments of the studied bacteria followed security and safety guidelines of Shandong University and the Second Hospital of Shandong University. All procedures were approved by the Scientific Ethics Committee of the Second Hospital of Shandong University with Approval No. KYLL-2020(LW)-044.

### Susceptibility Tests

Drug susceptibility testing was carried out by the disk diffusion method, and the standard for inhibition zones followed the Clinical and Laboratory Standards Institute (CLSI) guidelines (Clinical and Laboratory Standards Institute, 2018b). Minimum Inhibition Concentrations (MICs) for all antibiotics (ampicillin, amoxicillin-clavulanate, ceftazidime-avibactam, piperacillin-tazobactam, ampicillin-sulbactam, ticarcillin-clavulanate, cefoperazone, cefotaxime, ceftazidime, cefoxitin, cefepime, cefazolin, imipenem, meropenem, kanamycin, ciprofloxacin, gatifloxacin, nalidixic acid, chloramphenicol, trimethoprim, and tetracycline) but tigecycline was determined with the agar dilution method following CLSI guidelines (Clinical and Laboratory Standards Institute, 2019). For tigecycline, MIC was determined with the broth microdilution method following European Committee on Antimicrobial Susceptibility Testing (EUCAST) guidelines (Marchaim et al., 2014). *E*. *coli* ATCC 25922 was used as the control strain for most antibiotics. *E. coli* ATCC 27853 was used as the control strain for carbapenems. For resistance against β-lactam/BLI combinations, *E*. *coli* ATCC 35218 was used as the control strain as instructed by the CLSI guidelines (Clinical and Laboratory Standards Institute, 2018a,b, 2019).

### Whole Genome Sequencing and Sequence Analyses

The genomic DNA of *E. coli* W60 was extracted with the SDS method (Natarajan et al., 2016). Libraries for single-molecule real-time (SMRT) sequencing was constructed with an insert size of 10 kb using the SMRTbell^TM^ Template kit, version 1.0. Sequencing libraries were generated using NEBNext^TM^ Ultra^®^ DNA Library Prep Kit for Illumina (NEB, United States) following manufacturer’s recommendations and index codes were added to attribute sequences to each sample. The whole genome of *E. coli* W60 was sequenced using PacBio Sequel platform and Illumina NovaSeq PE150 at the Beijing Novogene Bioinformatics Technology Co., Ltd., SMRT Link v5.1.0 software was used^1^ for read assembly (Ardui et al., 2018; Reiner et al., 2018), which was further optimized by the Arrow software (part of SMRT Link v5.1.0). General function annotation databases GO (Gene Ontology, 2015), KEGG (Kanehisa and Goto, 2000), COG (Natale et al., 2000), NR (Li et al., 2002), Pfam (El-Gebali et al., 2019), TCDB (Saier et al., 2006), and Swiss-Prot (Boeckmann et al., 2003) were used for functional annotation of genes, and the Comprehensive Antibiotic Resistance Database (CARD) was used to manually annotate antimicrobial resistance genes (ARGs) (Jia et al., 2017). PlasmidFinder 2.1^2^ was used to analyze plasmid types (Carattoli et al., 2014).

### Mating Experiment

The ability of plasmids to transfer was determined by mating experiment, and the *E. coli* J53 was used as recipient strain. Transconjugants were selected on LB agar supplemented with different antibiotic agents: pECW601 was selected by LB agar containing NaN_3_ (100 μg/ml) and trimethoprim (30 μg/ml), pECW602 was selected by LB agar containing NaN_3_ (100 μg/ml), fosfomycin (50 μg/ml), and glucose 6-phosphate (25 μg/ml) according to CLSI standards (Clinical Laboratory Standards Institute, 2018b). Different antibiotics were used for screening transconjugants because pECW601 and pECW602 are, respectively, the only genetic determinants in the donor *E. coli* W60 strain that encode resistance for trimethoprim and fosfomycin as predicted by genomic analysis. Transconjugants were confirmed by amplifying the drug resistance genes and plasmid specific *rep* genes, followed by sequencing for final confirmation (Supplementary Figure S1). The sequences of primers for transconjugant confirmation are shown in Supplementary Table S1.

### Cloning of *bla*_NDM–5_ and *bla*_TEM–W60_

The *bla*_NDM–5_ and *bla*_TEM–W60_ genes were amplified and cloned into pBCKS(+). Primers used are shown in Supplementary Table S2.

### Accession Numbers

The nucleotide sequences of *E. coli* W60 genome and its plasmids can be found on NCBI under accession numbers CP058342, CP058343 and CP058344.

### Bioinformatics

SerotypeFinder 2.0^3^ was used for serotype prediction (Joensen et al., 2015), followed by serum aggregation reaction experiment for confirmation. Prediction of signal peptides was performed using SignalP 5.0^4^ (Armenteros et al., 2019). Sequence alignment was performed using ESPript 3.0^5^ with the Maximum Likelihood method and Poisson correction model (Robert and Gouet, 2014). Phylogenetic analysis was done by MEGA-X (Kumar et al., 2018). Protein structure analysis was performed using PyMol (Seeliger and de Groot, 2010).

### Ethics

All experiments in this work were performed adhering to the Declaration of Helsinki and were approved by the Scientific Ethics Committee of the Second Hospital of Shandong University with Approval No. KYLL-2020(LW)-044.

## Results

### Isolation and Resistance Properties of a Clinical XDR *E. coli* W60 Strain

*Escherichia coli* strain W60 was isolated from the urine sample of a bladder tumor patient from the Second Hospital of Shandong University. *In silico* prediction of the serotype was performed for *E. coli* W60, suggesting it was either serotype O101, O8, or H9. Further serum aggregation reaction assay was performed, showing that *E. coli* W60 belongs to serotype O101 (Supplementary Figure S2). O101 is a common enterotoxigenic *E. coli* (ETEC) serotype originated from pigs and cattle (Staaf et al., 1997). The antibiotic resistance profiles were determined for this strain by testing its resistance against major classes of antibiotics including β-lactams, quinolones, carbapenems, aminoglycosides, chloramphenicol, trimethoprim, tigecycline, macrolides, and polymyxins. *E. coli* W60 was found resistant to all antibiotics tested except for tigecycline (Table 1). Of particular interest, *E. coli* W60 is highly resistant to all commonly available β-lactam/BLI combinations with MIC values much higher than the resistance breakpoint.

**TABLE 1:** Antimicrobial resistance of *Escherichia coli* W60.

Antibiotic class	Antibiotics	Antimicrobial resistance^1^	
		Inhibition zone (mm)	MIC (mg/L)
β-lactam	Ampicillin (AMP)	*R*(0)	*R*(>512)
	Amoxicillin-clavulanate (AMC)	*R*(0)	*R*(64/32)
	Ceftazidime-avibactam (CAZ-AVI)	*R*(13)	*R*(>512/4)
	Piperacillin-tazobactam (TZP)	*R*(0)	*R*(>512/4)
	Ampicillin-sulbactam (SAM)	*R*(0)	*R*(>256/128)
	Ticarcillin-clavulanate (TIM)	*R*(7)	*R*(>256/2)
	Cefoperazone (CFP)	*R*(0)	*R*(>512)
	Cefotaxime (CTX)	*R*(0)	*R*(>512)
	Ceftazidime (CAZ)	*R*(0)	*R*(>512)
	Cefoxitin (FOX)	*R*(0)	*R*(>512)
	Cefepime (FEP)	*R*(0)	*R*(512)
	Cefazolin (CFZ)	*R*(0)	*R*(>512)
Carbapenem	Imipenem (IPM)	*R*(14)	*I*(2)
	Meropenem (MEM)	*R*(0)	*R*(4)
Aminoglycoside	Kanamycin (KAN)	*R*(12)	*R*(64)
Quinolone	Ciprofloxacin (CIP)	*R*(0)	*R*(32)
	Gatifloxacin (GAT)	*R*(9)	*R*(8)
	Nalidixic acid (NAL)	*R*(0)	*R*(512)
Phenicol	Chloramphenicol (CHL)	*R*(8)	*R*(256)
Diaminopyrimidine	Trimethoprim (TMP)	*R*(0)	*R*(>512)
Glycylcycline	Tigecycline (TGC)	*S*(18)	*S*(0.5)
Tetracycline	Tetracycline (TET)	*R*(10)	*R*(128)

*^1^R, resistant; I, intermediate; S, sensitive.*

### Genome Characteristics and Genotypic Basis for Antibiotic Resistance of *E. coli* W60

**FIGURE 1:**
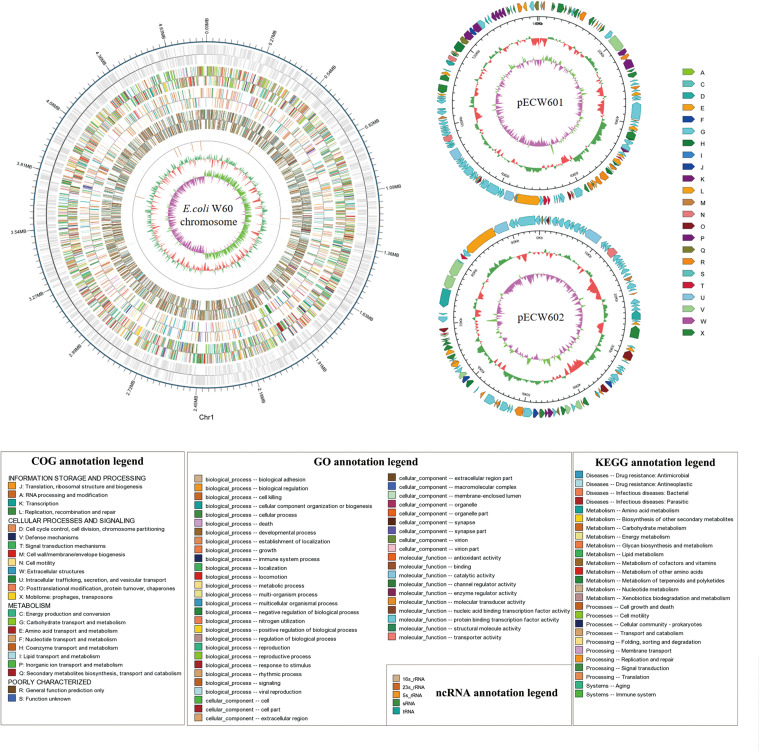
Whole genome map of pECW601, pECW602 and chromosome of *E. coli* W60. The outermost circle is the coordinates of the genomic feature. From outside to inside the circles indicates coding genes, gene function annotation results, ncRNAs, genome GC contents, genome GC skew values. For plasmids, from the outside to the inside the circles indicate COG functional annotation classification genes (clockwise arrow indicates positive strand coding), genome sequence position coordinates, genome GC content, genome GC skew values distribution. COG (KOG), KEGG, GO databases were used for gene annotation. Different colors represent different functions of genes. For genome GC content, the inward red part indicates that the GC content of the region is lower than the average GC content of the whole genome, and the outward green part indicates the opposite; for the genome GC skew value, the inward pink part indicates that in the region the G content is lower than the C content, and the outward light green part indicates the opposite.

Whole genome sequencing was performed with PacBio and Illumina sequencing on *E. coli* W60. *E. coli* W60 has a chromosome at the size of 4,808,792 bp and GC content of 50.8% (Figure 1). Two circular plasmids were identified from *E. coli* W60, respectively, named pECW601 and pECW602. BLAST analysis of both plasmids found no known plasmids that share both high sequence identity and coverage.

**FIGURE 2:**
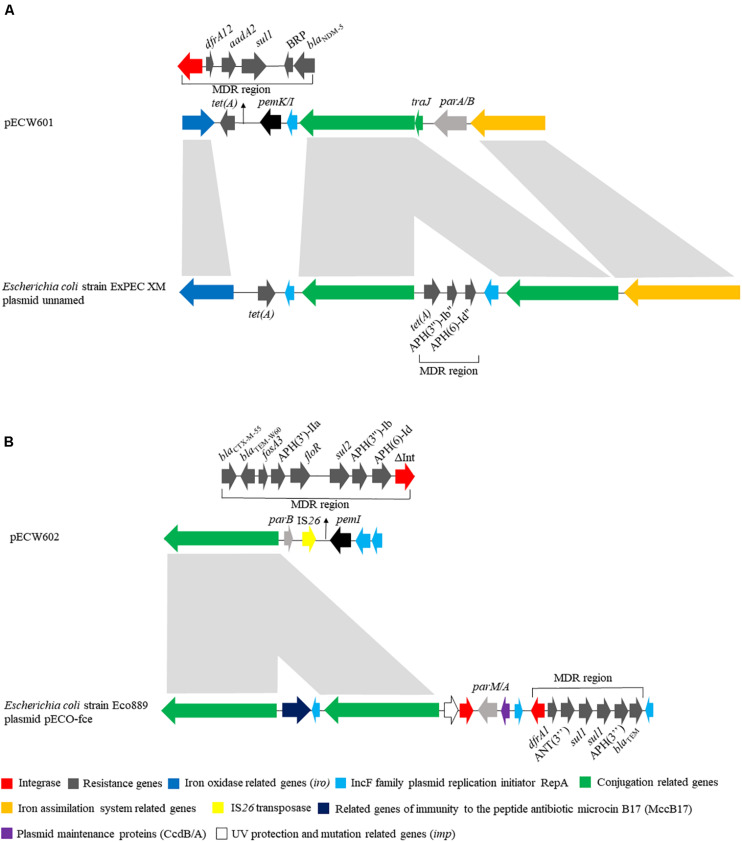
Linear schematic of sequence comparison between plasmids found in this work and their closest relatives. **(A)** Comparison between pECW601 and *Escherichia coli* strain ExPEC XM plasmid unnamed; **(B)** comparison between pECW602 and *E. coli* strain Eco889 plasmid pECO-fce. Different colors represent gene clusters with different functions. Arrows indicate the direction of genes. The light gray indicates high similarity between sequences.

pECW601 has a size of 140,410 bp. Its closest known relative is a *E. coli*-harboring unnamed plasmid from that has a size of 286,854 bp (GenBank accession number CP025329.1). Plasmid replication analysis showed that pECW601 is an IncFIB type plasmid. Comparison of pECW601 and CP025329.1 show that both plasmids share similar conjugation genes, iron oxidase related genes and IS*26* transposase genes but their MDR regions are fundamentally different (Figure 2A). In addition, CP025329.1 has two copies of the conjugation gene clusters but pECW601 has only one. Therefore, a conclusion can be made that pECW601 is a new multidrug resistance plasmid.

pECW602 has a size of 94,780 bp. Its closest known relative is pECO-fce from *E. coli* Eco889 (GenBank: CP015160.1). Plasmid replication analysis showed that pECW602 is an IncFII type plasmid. Both plasmids share the backbone conjugation gene cluster although CP015160.1 has 2 such clusters while pECW602 has only one. The MDR regions of the two plasmids have little in common, along with other minor different features between the two plasmids (Figure 2B). This comparison suggests that pECW602 is also a new plasmid.

Analysis of the genomic sequence of *E. coli* W60 reveals resistance determinants putatively responsible for the antimicrobial resistance phenotype of this strain. Two AmpC-type and one AmpH-type β-lactamases are encoded by the chromosome that are potentially responsible for resistance against β-lactams (Supplementary Table S3). The chromosome harbors a *gyrA* gene that encodes a D87N/S83L variant and a *pacC* gene that encodes a S80I variant (Supplementary Table S3). Both variants are responsible for resistance to quinolones (Yoshida et al., 1990). Respectively, 6 and 8 antimicrobial resistance genes (ARGs) were found on pECW601 and pECW602 (Table 2 and Supplementary Tables S4, S5). ARGs responsible for the resistance to aminoglycosides, β-lactams and sulfonamides are found on both plasmids. pECW601 harbors ARGs responsible for the resistance to trimethoprim, tetracycline and glycopeptides, while pECW602 harbors ARGs responsible for the resistance to chloramphenicol and fosfomycin. Therefore, genetic features were found for all the major resistant antibiotic classes investigated in the antimicrobial resistance phenotype analysis (Table 1), and the two multidrug resistance plasmids account for the resistance to most of these antibiotics.

**TABLE 2:** Presence of ARGs on plasmids.

Targeted antibiotic class	pECW601	pECW602
Aminoglycosides	*aadA2*	*APH(3′)-IIa*
		*APH(3′′)-Ib*
		*APH(6)-Id*
β-lactam	*bla*_NDM–5_	*bla*_CTX–M–55_
		*bla*_TEM–W60_
Diaminopyrimidine	*dfrA12*	
Sulfonamides	*sul1*	*sul2*
Phenicols		*floR*
Tetracycline	*tetA*	
Fosfomycin		*fosA3*
Glycopeptide	*ble*_MBL_	

Genetic analysis of the *E. coli* W60 genome leads to two interesting observations: *E. coli* W60 is resistant to all the β-lactam/BLI combinations tested, while the genetic basis for this observation remains unclear; pECW602 harbors a truncated version of *bla*_TEM–1_, leading us to wonder its role in mediating β-lactam resistance. Both these observations were further investigated in this work.

### Transferability of pECW601, pECW602, and β-Lactam/BLI Combination Resistance Phenotypes

Conjugation assays between *E. coli* W60 and the recipient *E. coli* J53 strain show that both pECW601 and pECW602 are transferable plasmids. Analysis of the antimicrobial resistance phenotypes of both transconjugants leads to the finding that the pECW601-harboring transconjugant showed nearly the same high level of resistance to β-lactam/BLI combination as *E. coli* W60 (Table 3). Considering the only β-lactamase-coding gene on pECW601 is *bla*_NDM–5_, a hypothesis is raised that *bla*_NDM–5_ can lead to high level resistance to β-lactam/BLI combinations in *E. coli*.

**TABLE 3:** Antibiotic sensitivity of pECW601 and pECW602-containing transconjugants.

Antibiotics^1^	W60^2^ (mg/L)	J53^3^ (mg/L)	J53/pECW601^4^ (mg/L)	J53/pECW602^5^ (mg/L)
AMP	>512	2	>512	>512
CFP	>512	<0.125	>512	128
CTX	>512	<0.125	>512	128
CAZ	>512	0.25	>512	16
FOX	>512	16	>512	32
FEP	512	<0.125	512	16
CFZ	>512	1	>512	>512
AMC	64/32	4/2	64/32	4/2
CAZ-AVI	>512/4	0.25/4	>256/4	0.125/4
TZP	>512/4	4/4	>256/4	4/4
SAM	>256/128	4/2	>256/128	16/8
TIM	>256/2	2/2	>256/2	16/2

*^1^AMP, ampicillin; AMC, amoxicillin-clavulanate; CAZ-AVI, ceftazidime-avibactam; TZP, piperacillin-tazobactam; SAM, ampicillin-sulbactam; TIM, ticarcillin-clavulanate; CFP, cefoperazone; CTX, cefotaxime; CAZ, ceftazidime; FOX, cefoxitin; FEP, cefepime; CFZ, cefazolin. ^2^W60, E. coli W60.^3^J53, E. coli J53.^4^J53/pECW601, E. coli J53 transconjugant harboring pECW601.^5^J53/pECW602, E. coli J53 transconjugant harboring pECW602.*

### β-Lactam/BLI Combination Resistance of *bla*_NDM–5_-Harboring *E. coli* Strain

To further explore the role of *bla*_NDM–5_ in the resistance of β-lactam related antibiotics, we cloned *bla*_NDM–5_ into pBCKS(+) plasmid and transformed the resulting construct to *E. coli* DH5α. An empty pBCKS(+) vector does not increase the resistance of *E. coli* DH5α to β-lactams or β-lactam/BLI combinations. However, *bla*_NDM–5_-containing pBCKS(+) increased the resistance of *E. coli* DH5α to β-lactams by 4–256-fold, and increased the resistance of *E. coli* DH5α to β-lactam/BLI combinations by 8–1,024-fold (Table 4). The finding that *bla*_NDM–5_ leads to high resistance to β-lactam/BLI combinations in *E. coli* DH5α, together with the observation that pECW601-containing *E. coli* J53 transconjugant is highly resistant to β-lactam/BLI combinations, experimentally confirm that *bla*_NDM–5_ leads to resistance to β-lactam/BLI combinations, and is the reason for the high level of resistance to β-lactam/BLI combinations of *E. coli* W60.

**TABLE 4:** Antibiotic sensitivity of *bla*_NDM–5_ and *bla*_NDM–5_/*ble*_MBL_-harboring strains.

Antibiotics^1^	DH5α^2^ (mg/L)	DH5α/pBCKS(+)^3^ (mg/L)	DH5α/pBCKS(+)-NDM5^4^ (mg/L)	DH5α/pBCKS(+)-NDM5+BLE^5^ (mg/L)
AMP	1	2	64	128
CFP	<0.125	<0.125	8	8
CTX	<0.125	<0.125	32	32
CAZ	<0.125	<0.125	64	128
FOX	16	16	64	256
FEP	<0.125	<0.125	1	1
CFZ	2	1	256	256
AMC	2/1	4/2	64/32	64/32
CAZ-AVI	<0.125/4	<0.125/4	128/4	128/4
TZP	2/4	4/4	16/4	16/4
SAM	2/1	2/1	64/32	128/64
TIM	1/2	2/2	256/2	256/2

*^1^AMP, ampicillin; AMC, amoxicillin-clavulanate; CAZ-AVI, ceftazidime-avibactam; TZP, piperacillin-tazobactam; SAM, ampicillin-sulbactam; TIM, ticarcillin-clavulanate; CFP, cefoperazone; CTX, cefotaxime; CAZ, ceftazidime; FOX, cefoxitin; FEP, cefepime; CFZ, cefazolin. ^2^DH5α, E. coli DH5α.^3^DH5α/pBCKS(+), E. coli DH5α containing empty pBCKS(+) vector.^4^DH5α/pBCKS(+)-NDM5, bla_NDM–5_-harboring E. coli DH5α strain.^5^DH5α/pBCKS(+)-NDM5 + BLE, bla_NDM–5_/ble_MBL_-harboring E. coli DH5α strain.*

A bleomycin resistance-conferring *ble*_MBL_ gene is located downstream of *bla*_NDM–5_ under the control of the same promoter on pECW601. The potential impact of this gene on the function of *bla*_NDM–5_ was probed by cloning both *bla*_NDM–5_ and *ble*_MBL_ to pBCKS(+), transforming the construct to *E. coli* DH5α, and comparing the resistance of the transformant to β-lactams and β-lactam/BLI combinations (Table 4). Increased resistance, although by only two–fourfold, was found for ampcilin, ceftazidime, cefoxitin, and ampicillin-sulbactam. This finding suggests a potential function of *ble*_MBL_ in enhancing the role of *bla*_NDM–5_ in β-lactam resistance.

### Presence and Function of a Truncated *bla*_TEM_ Gene on pECW602

**FIGURE 3:**
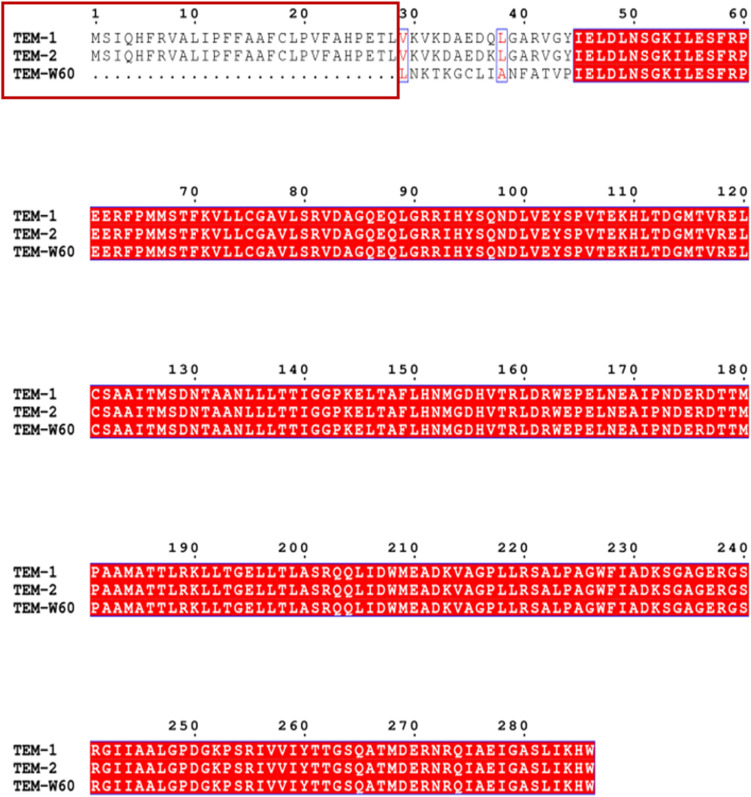
Multiple sequence alignment of TEM-W60, TEM1, and TEM2. Red box indicates the missing N-terminal region for TEM-W60. Blue box indicates mutations.

**FIGURE 4:**
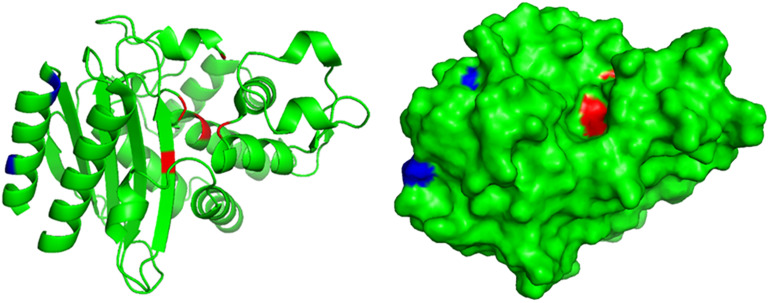
Structural analysis of TEM-W60. The structure of TEM was previously determined (PDB ID 1M40). Blue color indicates mutated amino acids in TEM-W60. Red color indicates key residues for the activity of TEM.

A truncated *bla*_TEM_ gene that encodes a TEM β-lactamase missing the N-terminal 28 amino acids, termed *bla*_TEM–W60_, was found on pECW602. Sequence comparison of TEM-W60 with TEM-1 and TEM-2 showed that other than missing the N-terminal 28 amino acids, TEM-W60 also has two mutations V29L and L38A (Figure 3). Phylogenetic analysis suggest that TEM-W60 does not cluster with known TEM β-lactamases (Supplementary Figure S3). The function of *bla*_TEM–W60_ was analyzed by cloning it into pBCKS(+), transforming the construct into *E. coli* DH5α, and analyzing the resistance of the transformant to β-lactams and β-lactam/BLI combinations (Table 5). It was found that *bla*_TEM–W60_ has little impact on resistance to β-lactams and β-lactam/BLI combinations. Structural analysis showed the two mutated amino acids reside on the N-terminal α-helix of TEM β-lactamase, far away from the active site and all other sites important for the activity of β-lactamase (Figure 4) (Jelsch et al., 1993; Minasov et al., 2002). It is therefore unlikely that these two substitutions significantly impact β-lactamase activity. Further sequence analysis predicted that the first 23 amino acids of TEM-1 form the signal peptide that is critical for secretion (Supplementary Figure S4). As β-lactamases are extracellular or periplasmic proteins and need to be secreted for their function (Livermore, 1995), it strongly suggests that the loss of function for TEM-W60 is due to the loss of a signal peptide and subsequent inability for secretion.

**TABLE 5:** Antibiotic sensitivity of *bla*_TEM–W60_-harboring strains.

Antibiotics^1^	DH5α^2^ (mg/L)	DH5α/pBCKS(+)^3^ (mg/L)	DH5α/pBCKS(+)-TEM-W60^4^ (mg/L)
AMP	1	2	2
CFP	<0.125	<0.125	<0.125
CTX	<0.125	<0.125	<0.125
CAZ	<0.125	<0.125	<0.125
FOX	16	16	32
FEP	<0.125	<0.125	<0.125
CFZ	2	1	1
AMC	2/1	4/2	4/2
CAZ-AVI	<0.125/4	<0.125/4	<0.125/4
TZP	2/4	4/4	4/4
SAM	2/1	2/1	2/1
TIM	1/2	2/2	2/2

*^1^AMP, ampicillin; AMC, amoxicillin-clavulanate; CAZ-AVI, ceftazidime-avibactam; TZP, piperacillin-tazobactam; SAM, ampicillin-sulbactam; TIM, ticarcillin-clavulanate; CFP, cefoperazone; CTX, cefotaxime; CAZ, ceftazidime; FOX, cefoxitin; FEP, cefepime; CFZ, cefazolin. ^2^DH5α, E. coli DH5α.^3^DH5α/pBCKS(+), E. coli DH5α containing empty pBCKS(+) vector.^4^DH5α/PBCKS(+)-TEM-W60, bla_TEM–W60_-harboring E. coli DH5α strain.*

## Discussion

MDR and XDR pathogenic bacteria pose a significant threat to human health (Karaiskos and Giamarellou, 2014). Due to their resistance to multiple antibiotic agents, the treatment of postoperative infections becomes difficult, thus increasing the morbidity and mortality of patients. Understanding the underlying drug resistance mechanisms of these pathogens helps us finding solutions for the long-standing antibiotic resistance problem. For instance, β-lactamase inhibitors were developed to inhibit β-lactamases that caused β-lactam resistance.

In this study, we identified an XDR *E. coli* W60 strain in the urine sample of a patient with postoperative infection. *E. coli* W60 belongs to serotype O101 after analysis by agglutination reaction assay. Unlike the enterohaemorrhagic *E. coli* O157, which is highly pathogenic and virulent, although serotype O101 has been shown to be related to diarrhea and urinary tract infection (Mandal et al., 2001; Sun et al., 2019), it is only a risk factor and has no direct connection with human disease. This strain shows resistance to almost all common antibiotic agents, including β-lactams, aminoglycosides, carbapenems, quinolones and etc. Whole genome sequencing shows that *E. coli* W60 contains two new multidrug resistance plasmids, pECW601 and pECW602. Analysis of the ARGs harbored by these two plasmids suggested that these plasmids are the primary reason for the extensively-drug resistance phenotype, while resistance genes located into the chromosomes are presumably responsible for only β-lactam and quinolone resistance. Further conjugation assays show both plasmids are transferable. These findings again confirm the danger of multidrug resistance plasmids: the concentration of different multidrug resistance plasmids into one bacterium can lead to the generation of highly resistant pathogens as demonstrated in this work and the work of others (Zhao et al., 2010; Guo et al., 2017; Li et al., 2019). Because transfer of plasmids is way more efficient than the evolution of new antibiotic resistance genotypes, we suspect this is the primary route for the generation of extensively- or pan-drug resistant pathogens. The danger of multidrug resistance plasmids should therefore be given high attention. The fact that both multidrug resistance plasmids found in this work are new rings a bell for us: there could be many more such plasmids out there waiting to be found. We therefore would like to call upon scientists and doctors in the field of antimicrobial resistance to perform more surveillance studies on clinical multidrug resistance plasmids and have a better understanding on the types and structures of these mobile genetic elements.

A particularly interesting and troubling feature of *E. coli* W60 is that it is resistant to all the β-lactam/BLI combinations tested. Further genetic analysis shows that the *bla*_NDM–5_ gene harbored by pECW601 is the reason for the resistance of β-lactam/BLI combinations. β-lactams are by far the most important antibiotics for their high efficiency to both Gram-positive and Gram-negative bacteria, and for their relatively better safety to human in comparison with other more recently introduced last-resort antibiotics (Bush and Bradford, 2016). Therefore, reusing β-lactams that already develop widespread resistance by combining BLIs is a great strategy and the first choice when treating infections of β-lactam resistant pathogens. It has been long suspected that this strategy does not work well with MBLs for their different resistance mechanism (Bebrone, 2007). This work provides solid microbiological and genetic evidence that NDM-5 can lead to high β-lactam/BLI resistance against all commonly available β-lactam/BLI combinations, and it is already causing strong resistance in a clinical pathogen. The mechanism behind this phenotype is likely that these commonly used BLIs (tazobactam, clavulanate, sulbactam, and avibactam) are serine-β-lactamases inhibitors that inhibit the serine active site of β-lactamase, but are ineffective against the zinc ion-containing active sites for MBLs (Docquier and Mangani, 2018). It needs to be pointed out that the β-lactamases besides *bla*_NDM–5_ encoded by *E. coli* W60 also contribute to this β-lactam/BLI combination resistance phenotype, as *E. coli* DH5α harboring only *bla*_NDM–5_ showed a weaker resistance in comparison with *E. coli* W60 or pECW601-containing *E. coli* J53. The finding that *bla*_NDM–5_ confers widespread resistance to β-lactam/BLI combinations again confirms the danger of MBLs, as they render β-lactams, β-lactam/BLIs, and carbapenems (in other words all β-lactam related antibiotics) ineffective. Susceptible testing showed that *E. coli* DH5α containing both *bla*_NDM–5_ and *ble*_MBL_ is slightly more resistant to ampicillin, ceftazidime, cefoxitin, and ampicillin-sulbactam than *E. coli* DH5α containing only *bla*_NDM__–__5_ (Table 4). *ble*_MBL_ encodes a BRP protein that exerts resistance to bleomycin by specifically binding to bleomycin family antibiotics, but does not appear to interact with β-lactams or β-lactamases. Previous research reported that *ble*_MBL_ and *bla*_NDM_ genes are often co-transcribed, and suggested that BRP influences *E. coli* mutation rates to stabilize NDM resistance traits (Dortet et al., 2012). An earlier report suggested that the existence of bleomycin resistance phenotype protects bacteria from external DNA damage through the DNA repair system, thereby conferring better fitness of bacteria and facilitating the inheritance of genetic characteristics (Blot et al., 1991). The enhancement of β-lactam resistance by *ble*_MBL_ found in this work could be for the same reason, and this new role of *ble*_MBL_ in β-lactam resistance makes more sense for the frequently observed co-transcription of *ble*_MBL_ and *bla*_NDM_.

A survey of other β-lactamase genes leads to the finding of a truncated *bla*_TEM_ gene on pECW602 that encode a TEM β-lactamase with 28 amino acids deleted at the N-terminus. This gene was found unfunctional presumably due to the loss of the signal peptide coding region in comparison with other *bla*_TEM_ genes. This finding confirms the importance of the signal peptide for TEM β-lactamase, understandably for its critical role in β-lactamase secretion.

## Conclusion

Combining genomic, microbiological and genetic approaches, we identified the genetic basis for the extensively-drug resistance phenotype of the clinical *E. coli* W60 strain. Two new conjugative multi-resistance plasmids pECW601 and pECW602 were found in *E. coli* W60, and were confirmed to be the primary determinants of the extensively drug resistance phenotype. Resistance phenotype analysis showed that *E. coli* W60 is resistant to all commonly available β-lactam/BLI combinations. Further genetic analysis showed that the NDM-5 β-lactamase coded on pECW601 is responsible for this phenotype, which is further enhanced by co-expressing BRP. A new unfunctional truncated TEM β-lactamase that lacks the signal peptide-containing N-terminus is encoded by pECW602, suggesting the critical role of the signal peptide on the function of β-lactamases. Findings in this work shows the danger of transferable multidrug resistance plasmids and metallo-β-lactamases. We hope with this work these dangers are given enough attention in further developing methods for containing antimicrobial resistance.

## Data Availability Statement

The datasets presented in this study can be found in online repositories. The names of the repository/repositories and accession number(s) can be found below: NCBI BioProject (accession: PRJNA642190).

## Author Contributions

WW, LL, MZ, ZL, WS, and FL performed microbial and genetic experiments. YN, TL, and XZ isolated bacteria. MW and WW performed bioinformatic analysis. MW, WW, XZ, and HX analyzed the data. MW, WW, XZ, and HX wrote the manuscript. MW, XZ, and HX conceived of the study and oversaw the project. All authors read and approved the manuscript.

## Conflict of Interest

FL was employed by the company Shandong Shian Chemical Co., Ltd., Dezhou, China. The remaining authors declare that the research was conducted in the absence of any commercial or financial relationships that could be construed as a potential conflict of interest.

## Footnotes

1 https://www.pacb.com/support/software-downloads/

2 https://cge.cbs.dtu.dk/services/PlasmidFinder/

3 https://cge.cbs.dtu.dk/services/SerotypeFinder/

4 http://www.cbs.dtu.dk/services/SignalP/

5 http://espript.ibcp.fr/ESPript/cgi-bin/ESPript.cgi
