# Supplementary material for: A Clinical Extensively-Drug Resistant (XDR) Escherichia coli and Role of Its β-Lactamase Genes
Source: Front Microbiol. 2020 Dec 10;11:590357. doi: 10.3389/fmicb.2020.590357 (PMC7758502; doi:10.3389/fmicb.2020.590357)
Supplement: Supplementary file 1 [file Data_Sheet_1.PDF]

Supplementary Material:

A clinical extensively-drug resistant (XDR) *Escherichia coli* and role of its β-lactamase genes

Mingyu Wang[1, a], Wenjia Wang[1, a], Yu Niu[2], Ting Liu[2], Ling Li[1], Mengge Zhang[1], Ziyun Li[1], Wenya Su[1], Fangyue Liu[3], Xuhua Zhang[2, *], Hai Xu[1, *]

[1]State Key Laboratory of Microbial Technology, Microbial Technology Institute, Shandong University, Qingdao, China
[2]Laboratory Medicine Center, The Second Hospital of Shandong University, Jinan, China
[3]Shandong Shian Chemical Co., Ltd., Dezhou, China

***Correspondence:**
Xuhua Zhang
chh8105@126.com
Hai Xu
haixu@sdu.edu.cn;

[a] These authors contribute equally to this work.

1 SUPPLEMENTARY DATA

2 SUPPLEMENTARY TABLES AND FIGURES

2.1 Figures

Figure S1. PCR verification of transconjugants. For J53/pECW601, the *repA* gene of IncFIB plasmid and the unique *dfrA* and *tetA* genes on pECW601 were amplified; for J53/pECW602, the *repA* gene of IncFII plasmid and the unique *floR* and *fosA* genes on pECW602 were amplified. Amplified bands were verified by sequencing. The negative control used water as a template.

Figure S2. Serum agglutination of *E. coli* W60. (a) Agglutination of W60 and *Escherichia coli* diagnostic serum O101. A single colony of W60 was picked from the plate and mixed with 0.9% NaCl to prepare a suspension, and a drop of diagnostic serum O101 was added dropwise. Particle precipitation was occurred within one minute. (b) Mixture of W60 and 0.9% normal saline. Using 0.9% saline instead of diagnostic serum O101 as a negative control.

Figure S3. Phylogenetic tree of TEM family proteins. Thirty-two TEM protein variants and TEM-W60 for evolutionary relationship analysis was performed. Numbers at each node are bootstrap values. Bar, evolutionary distances.

Figure S4. Predicted signal peptides of TEM-1 and TEM-W60.

2.2 Tables

Table S1. Primers for verify the conjugation transfer of pECW601 and pECW602.

Primer name	Sequence	Target gene	Product size (bp)	Reference
IncFIB	**F:** GGAGTTCTGACACACGATTTTCTG<br>**R:** CTCCCGTCGCTTCAGGGCATT	IncFIB *repA*	702	(Carattoli A, et al.,2005)
IncFII	**F:** TGTCAGCTGCCGTTTCACCA<br>**R:** GTGCAGGATGGTGTGACTGA	IncFII *repA*	738	This study
dfrA	**F:** ATCTCGTTGCTGCGATGGGA<br>**R:** TGGGTGTACGGAATTACAGC	*dfrA12* in pECW601	445	This study
tetA	**F:** ATAGAAGCCGCATAGATCGC<br>**R:** GGCGGTCTTCTTCATCATGC	*tetA* in pECW601	441	This study
floR	**F:** GTCACGATCATTACAAGCGCG<br>**R:** TTCGTCATTGCGTCTCTGGG	*floR* in pECW602	527	This study
fosA	**F:** GGGATTGAATCATCTGACGC<br>**R:** AGTAAGACGCCCCTTCACT	*fosA3* in pECW602	299	This study

Table S2. Primers used for cloning in this work. a: Length includes TEM gene and possible promoter fragments upstream.

Gene	Primer	Tm	Product size (bp)	Reference
bla$_{\text{NDM5}}$	**F:**CCCAAGCTTATGGAATTGCCCAATATTATGCACCCGG<br>**R:**CGCGGATCCTCAGCGCAGCTTGTCGG	65℃	831	This study
bla$_{\text{TEM}}$	**F:**CCCAAGCTTTCTTCTTCCGCCACGCTG<br>**R:**TGCTCTAGATTACCAATGCTTAATCAGTGAGGCACC	63℃	2117	This study
bla$_{\text{NDM5}}$ +*ble*$_{\text{MBL}}$	**F:**CCCAAGCTTATGGAATTGCCCAATATTATGCACCCGG<br>**R:**CGCGGATCCTCAGTCGGGGTTCTGGATCA	65℃	1200	This study

Table S3. Annotation of *Escherichia coli* W60 chromosomal ARGs. Resistance Gene Identifier (RGI) of the Comprehensive Antibiotic Resistance Database (CARD) was used for annotation.

RGI Criteria	ARO Term	SNP	Detection Criteria	AMR Gene Family	Drug Class	Resistance Mechanism	% Identity of Matching Region	% Length of Reference Sequence
Perfect	*gadX*		protein homolog model	resistance-nodulation-cell division (RND) antibiotic efflux pump	macrolide antibiotic, fluoroquinolone antibiotic, penam	antibiotic efflux	100.0	100.00
Perfect	*mdtF*		protein homolog model	resistance-nodulation-cell division (RND) antibiotic efflux pump	macrolide antibiotic, fluoroquinolone antibiotic, penam	antibiotic efflux	100.0	100.00
Perfect	*mdtE*		protein homolog model	resistance-nodulation-cell division (RND) antibiotic efflux pump	macrolide antibiotic, fluoroquinolone antibiotic, penam	antibiotic efflux	100.0	100.00
Perfect	AcrF		protein homolog model	resistance-nodulation-cell division (RND) antibiotic efflux pump	fluoroquinolone antibiotic, cephalosporin, cephamycin, penam	antibiotic efflux	100.0	100.00
Perfect	*bacA*		protein homolog model	undecaprenyl pyrophosphate related proteins	peptide antibiotic	antibiotic target alteration	100.0	100.00
Perfect	*emrB*		protein homolog model	major facilitator superfamily (MFS)	fluoroquinolone	antibiotic efflux	100.0	100.00

			antibiotic efflux pump	antibiotic			
Perfect	*emrA*	protein homolog model	major facilitator superfamily (MFS) antibiotic efflux pump	fluoroquinolone antibiotic	antibiotic efflux	100.0	100.00
Perfect	*acrD*	protein homolog model	resistance-nodulation-cell division (RND) antibiotic efflux pump	aminoglycoside antibiotic	antibiotic efflux	100.0	100.00
Perfect	*Escherichia coli ampC1* beta-lactamase	protein homolog model	ampC-type beta-lactamase	cephalosporin, penam	antibiotic inactivation	100.0	100.00
Perfect	*evgA*	protein homolog model	major facilitator superfamily (MFS) antibiotic efflux pump, resistance-nodulation-cell division (RND) antibiotic efflux pump	macrolide antibiotic, fluoroquinolone antibiotic, penam, tetracycline antibiotic	antibiotic efflux	100.0	100.00
Perfect	*emrY*	protein homolog model	major facilitator superfamily (MFS) antibiotic efflux pump	tetracycline antibiotic	antibiotic efflux	100.0	100.00
Perfect	PmrF	protein homolog model	pmr phosphoethanolamine transferase	peptide antibiotic	antibiotic target alteration	100.0	100.00
Perfect	*baeS*	protein homolog model	resistance-nodulation-cell division (RND) antibiotic efflux pump	aminoglycoside antibiotic, aminocoumarin	antibiotic efflux	100.0	100.00

				antibiotic			
Perfect	*mdtB*	protein homolog model	resistance-nodulation-cell division (RND) antibiotic efflux pump	aminocoumarin antibiotic	antibiotic efflux	100.0	100.00
Perfect	*marA*	protein homolog model	resistance-nodulation-cell division (RND) antibiotic efflux pump, General Bacterial Porin with reduced permeability to beta-lactams	fluoroquinolone antibiotic, monobactam, carbapenem, cephalosporin, glycylcycline, cephamycin, penam, tetracycline antibiotic, rifamycin antibiotic, phenicol antibiotic, triclosan, penem	antibiotic efflux, reduced permeability to antibiotic	100.0	100.00
Perfect	H-NS	protein homolog model	major facilitator superfamily (MFS) antibiotic efflux pump, resistance-nodulation-cell division (RND) antibiotic efflux pump	macrolide antibiotic, fluoroquinolone antibiotic, cephalosporin, cephamycin, penam, tetracycline antibiotic	antibiotic efflux	100.0	100.00
Perfect	*mdtH*	protein homolog model	major facilitator superfamily (MFS) antibiotic efflux pump	fluoroquinolone antibiotic	antibiotic efflux	100.0	100.00
Perfect	*mdtG*	protein homolog model	major facilitator superfamily (MFS) antibiotic efflux pump	fosfomycin	antibiotic efflux	100.0	100.00

Perfect	*msbA*	protein homolog model	ATP-binding cassette (ABC) antibiotic efflux pump	nitroimidazole antibiotic	antibiotic efflux	100.0	100.00
Perfect	*kdpE*	protein homolog model	kdpDE	aminoglycoside antibiotic	antibiotic efflux	100.0	100.00
Perfect	*Escherichia coli acrA*	protein homolog model	resistance-nodulation-cell division (RND) antibiotic efflux pump	fluoroquinolone antibiotic, cephalosporin, glycylcycline, penam, tetracycline antibiotic, rifamycin antibiotic, phenicol antibiotic, triclosan	antibiotic efflux	100.0	100.00
Perfect	*acrB*	protein homolog model	resistance-nodulation-cell division (RND) antibiotic efflux pump	fluoroquinolone antibiotic, cephalosporin, glycylcycline, penam, tetracycline antibiotic, rifamycin antibiotic, phenicol antibiotic, triclosan	antibiotic efflux	100.0	100.00
Perfect	*Escherichia coli ampH* beta-lactamase	protein homolog model	ampC-type beta-lactamase	cephalosporin, penam	antibiotic inactivation	100.0	100.00
Perfect	*mdtN*	protein	major facilitator	nucleoside antibiotic,	antibiotic efflux	100.0	100.00

		homolog model	superfamily (MFS) antibiotic efflux pump	acridine dye			
Perfect	*mdtP*	protein homolog model	major facilitator superfamily (MFS) antibiotic efflux pump	nucleoside antibiotic, acridine dye	antibiotic efflux	100.0	100.00
Perfect	*cpxA*	protein homolog model	resistance-nodulation-cell division (RND) antibiotic efflux pump	aminoglycoside antibiotic, aminocoumarin antibiotic	antibiotic efflux	100.0	100.00
Strict	CRP	protein homolog model	resistance-nodulation-cell division (RND) antibiotic efflux pump	macrolide antibiotic, fluoroquinolone antibiotic, penam	antibiotic efflux	99.05	100.00
Strict	TolC	protein homolog model	ATP-binding cassette (ABC) antibiotic efflux pump, major facilitator superfamily (MFS) antibiotic efflux pump, resistance-nodulation-cell division (RND) antibiotic efflux pump	macrolide antibiotic, fluoroquinolone antibiotic, aminoglycoside antibiotic, carbapenem, cephalosporin, glycylcycline, cephamycin, penam, tetracycline antibiotic, peptide antibiotic, aminocoumarin antibiotic, rifamycin antibiotic, phenicol antibiotic, triclosan,	antibiotic efflux	100.0	99.60

				penem			
Strict	*emrR*	protein homolog model	major facilitator superfamily (MFS) antibiotic efflux pump	fluoroquinolone antibiotic	antibiotic efflux	94.43	100.00
Strict	*evgS*	protein homolog model	major facilitator superfamily (MFS) antibiotic efflux pump, resistance-nodulation-cell division (RND) antibiotic efflux pump	macrolide antibiotic, fluoroquinolone antibiotic, penam, tetracycline antibiotic	antibiotic efflux	99.25	100.00
Strict	*emrK*	protein homolog model	major facilitator superfamily (MFS) antibiotic efflux pump	tetracycline antibiotic	antibiotic efflux	100.0	110.26
Strict	*YojI*	protein homolog model	ATP-binding cassette (ABC) antibiotic efflux pump	peptide antibiotic	antibiotic efflux	98.72	100.00
Strict	*baeR*	protein homolog model	resistance-nodulation-cell division (RND) antibiotic efflux pump	aminoglycoside antibiotic, aminocoumarin antibiotic	antibiotic efflux	99.58	100.00
Strict	*mdtC*	protein homolog model	resistance-nodulation-cell division (RND) antibiotic efflux pump	aminocoumarin antibiotic	antibiotic efflux	99.61	100.00
Strict	*mdtA*	protein homolog model	resistance-nodulation-cell division (RND) antibiotic efflux pump	aminocoumarin antibiotic	antibiotic efflux	99.04	109.64

Strict	*Klebsiella pneumoniae* KpnE	protein homolog model	major facilitator superfamily (MFS) antibiotic efflux pump	macrolide antibiotic, aminoglycoside antibiotic, cephalosporin, tetracycline antibiotic, peptide antibiotic, rifamycin antibiotic	antibiotic efflux	82.2	100.83
Strict	*Klebsiella pneumoniae* KpnF	protein homolog model	major facilitator superfamily (MFS) antibiotic efflux pump	macrolide antibiotic, aminoglycoside antibiotic, cephalosporin, tetracycline antibiotic, peptide antibiotic, rifamycin antibiotic	antibiotic efflux	84.4	100.00
Strict	*Escherichia coli mdfA*	protein homolog model	major facilitator superfamily (MFS) antibiotic efflux pump	tetracycline antibiotic, benzalkonium chloride, rhodamine	antibiotic efflux	97.07	100.00
Strict	*mdtM*	protein homolog model	major facilitator superfamily (MFS) antibiotic efflux pump	fluoroquinolone antibiotic, lincosamide antibiotic, nucleoside antibiotic, acridine dye, phenicol antibiotic	antibiotic efflux	97.07	100.00
Strict	*Escherichia coli ampC* beta-lactamase	protein homolog model	ampC-type beta-lactamase	cephalosporin, penam	antibiotic inactivation	96.82	100.00

Strict	*eptA*		protein homolog model	pmr phosphoethanolamine transferase	peptide antibiotic	antibiotic target alteration	99.82	100.00
Strict	*mdtO*		protein homolog model	major facilitator superfamily (MFS) antibiotic efflux pump	nucleoside antibiotic, acridine dye	antibiotic efflux	99.85	100.00
Strict	*Escherichia coli* EF-Tu mutants conferring resistance to Pulvomycin	R234F	protein variant model	elfamycin resistant EF-Tu	elfamycin antibiotic	antibiotic target alteration	99.75	96.33
Strict	*Escherichia coli parC* conferring resistance to fluoroquinolone	S80I	protein variant model	fluoroquinolone resistant *parC*	fluoroquinolone antibiotic	antibiotic target alteration	99.73	100.00
Strict	*Escherichia coli gyrA* conferring resistance to fluoroquinolones	D87N, S83L	protein variant model	fluoroquinolone resistant *gyrA*	fluoroquinolone antibiotic	antibiotic target alteration	99.77	100.00
Strict	*Haemophilus influenzae*	D350N,	protein variant model	Penicillin-binding protein mutations conferring	cephalosporin,	antibiotic target alteration	52.56	97.05

	PBP3 conferring resistance to beta-lactam antibiotics	S357N		resistance to beta-lactam antibiotics	cephamycin, penam			
Strict	*Escherichia coli* EF-Tu mutants conferring resistance to Pulvomycin	R234F	protein variant model	elfamycin resistant EF-Tu	elfamycin antibiotic	antibiotic target alteration	99.75	96.33
Strict	*Escherichia coli marR* mutant conferring antibiotic resistance		protein overexpression model	resistance-nodulation-cell division (RND) antibiotic efflux pump	fluoroquinolone antibiotic, cephalosporin, glycylcycline, penam, tetracycline antibiotic, rifamycin antibiotic, phenicol antibiotic, triclosan	antibiotic target alteration, antibiotic efflux	100.0	100.00
Strict	*Escherichia coli acrR* with mutation conferring multidrug antibiotic		protein overexpression model	resistance-nodulation-cell division (RND) antibiotic efflux pump	fluoroquinolone antibiotic, cephalosporin, glycylcycline, penam, tetracycline antibiotic, rifamycin antibiotic, phenicol antibiotic,	antibiotic target alteration, antibiotic efflux	98.44	105.58

	resistance			triclosan			
Strict	*Escherichia coli soxR* with mutation conferring antibiotic resistance	protein overexpression model	ATP-binding cassette (ABC) antibiotic efflux pump, major facilitator superfamily (MFS) antibiotic efflux pump, resistance-nodulation-cell division (RND) antibiotic efflux pump	fluoroquinolone antibiotic, cephalosporin, glycylcycline, penam, tetracycline antibiotic, rifamycin antibiotic, phenicol antibiotic, triclosan	antibiotic target alteration, antibiotic efflux	99.35	100.00
Strict	*Escherichia coli soxS* with mutation conferring antibiotic resistance	protein overexpression model	ATP-binding cassette (ABC) antibiotic efflux pump, major facilitator superfamily (MFS) antibiotic efflux pump, resistance-nodulation-cell division (RND) antibiotic efflux pump, General Bacterial Porin with reduced permeability to beta-lactams	fluoroquinolone antibiotic, monobactam, carbapenem, cephalosporin, glycylcycline, cephamycin, penam, tetracycline antibiotic, rifamycin antibiotic, phenicol antibiotic, triclosan, penem	antibiotic target alteration, antibiotic efflux, reduced permeability to antibiotic	100.0	100.00

Table S4. Annotation pECW601 ARGs. RGI of CARD was used for annotation.

RGI Criteria	ARO Term	Detection Criteria	AMR Gene Family	Drug Class	Resistance Mechanism	% Identity of Matching Region	% Length of Reference Sequence
Perfect	*dfrA12*	protein homolog model	trimethoprim resistant dihydrofolate reductase *dfr*	diaminopyrimidine antibiotic	antibiotic target replacement	100.0	100.00
Perfect	*aadA2*	protein homolog model	ANT(3")	aminoglycoside antibiotic	antibiotic inactivation	100.0	100.00
Perfect	*sul1*	protein homolog model	sulfonamide resistant *sul*	sulfonamide antibiotic	antibiotic target replacement	100.0	100.00
Perfect	BRP(MBL)	protein homolog model	Bleomycin resistant protein	glycopeptide antibiotic	antibiotic inactivation	100.0	100.00
Perfect	NDM-5	protein homolog model	NDM beta-lactamase	carbapenem, cephalosporin, cephamycin, penam	antibiotic inactivation	100.0	100.00
Strict	*tet(A)*	protein homolog model	major facilitator superfamily (MFS) antibiotic efflux pump	tetracycline antibiotic	antibiotic efflux	99.74	94.10

Table S5. Annotation pECW602 ARGs. RGI of CARD was used for annotation.

RGI Criteria	ARO Term	Detection Criteria	AMR Gene Family	Drug Class	Resistance Mechanism	% Identity of Matching Region	% Length of Reference Sequence
Perfect	CTX-M-55	protein homolog model	CTX-M beta-lactamase	cephalosporin	antibiotic inactivation	100.0	100.00
Perfect	FosA3	protein homolog model	fosfomycin thiol transferase	fosfomycin	antibiotic inactivation	100.0	100.00
Perfect	APH(3')-IIa	protein homolog model	APH(3')	aminoglycoside antibiotic	antibiotic inactivation	100.0	100.00
Perfect	*sul2*	protein homolog model	sulfonamide resistant sul	sulfonamide antibiotic	antibiotic target replacement	100.0	100.00
Perfect	APH(3")-Ib	protein homolog model	APH(3")	aminoglycoside antibiotic	antibiotic inactivation	99.63	100.00
Strict	APH(6)-Id	protein homolog model	APH(6)	aminoglycoside antibiotic	antibiotic inactivation	99.64	100.00
Strict	TEM-2	protein homolog model	TEM beta-lactamase	Monobactam, penem, penam, cephalosporin	antibiotic inactivation	100.0	90.21
Strict	*floR*	protein homolog model	Major facilitator superfamily (MFS) antibiotic efflux pump	Rifamycin antibiotic, penam, cephalosporin, Fosfomycin, lincosamide antibiotic, peptide antibiotic,	Antibiotic efflux	99.26	100.00

	bicyclomycin, glycylcycline, acridinedye, benzalkonium chloride, tetracycline antibiotic, phenicol antibiotic, diaminopyrimidine antibiotic, isoniazid, macrolide antibiotic, oxazolidinone antibiotic, nitroimidazole antibiotic, nucleoside antibiotic, rhodamine, fluoroquinolone antibiotic, antibacterial free fatty acids

Reference

Carattoli A, Bertini A, Villa L, Falbo V, Hopkins KL, Threlfall EJ. Identification of plasmids by PCR-based replicon typing. J Microbiol Methods (2005) 63(3):219-28.
