# Supplementary figures and images for: A Clinical Extensively-Drug Resistant (XDR) Escherichia coli and Role of Its β-Lactamase Genes
Source: Front Microbiol. 2020 Dec 10;11:590357. doi: 10.3389/fmicb.2020.590357 (PMC7758502; doi:10.3389/fmicb.2020.590357)

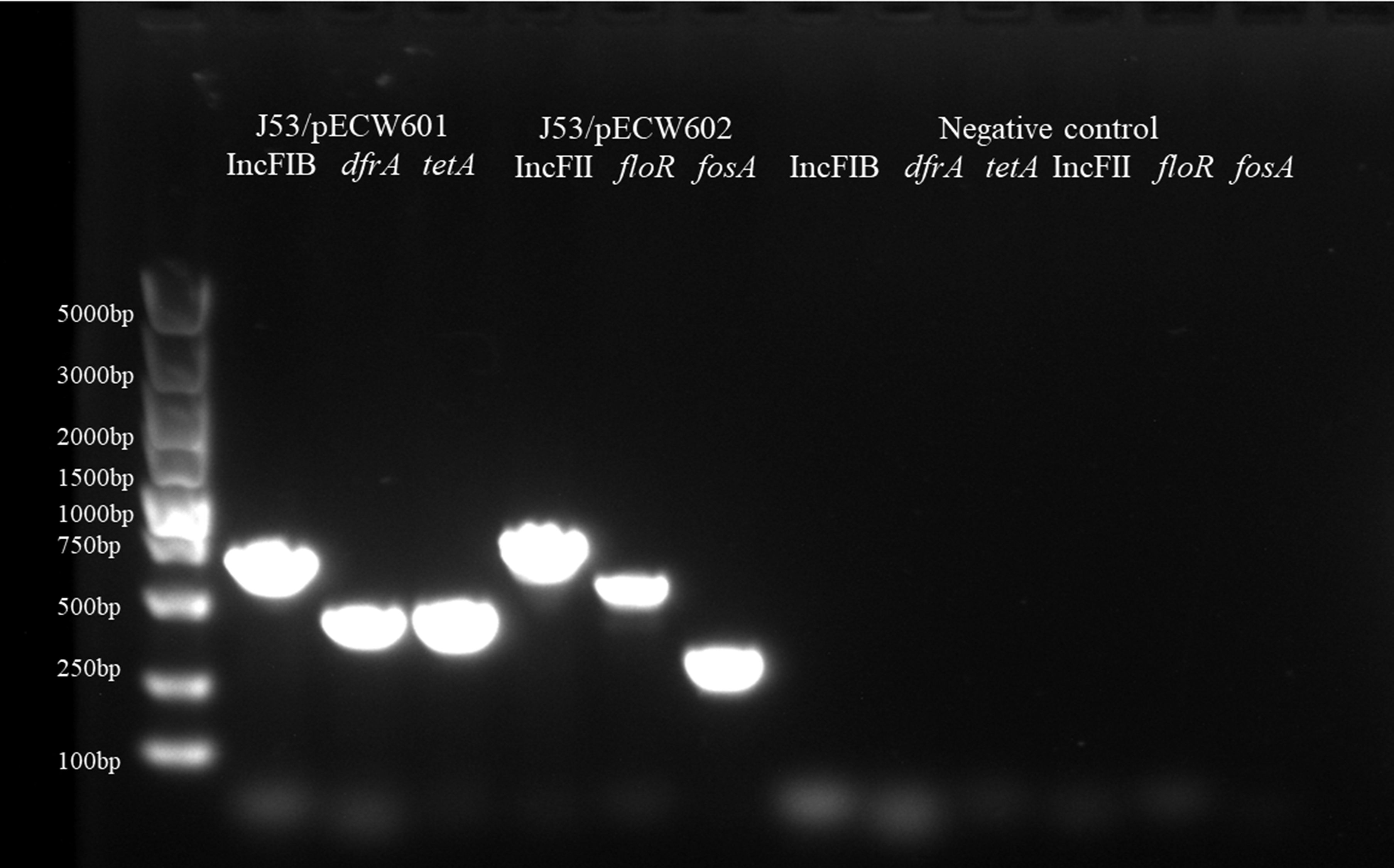

Supplement: Supplementary file 2 [file Image_1.TIF]

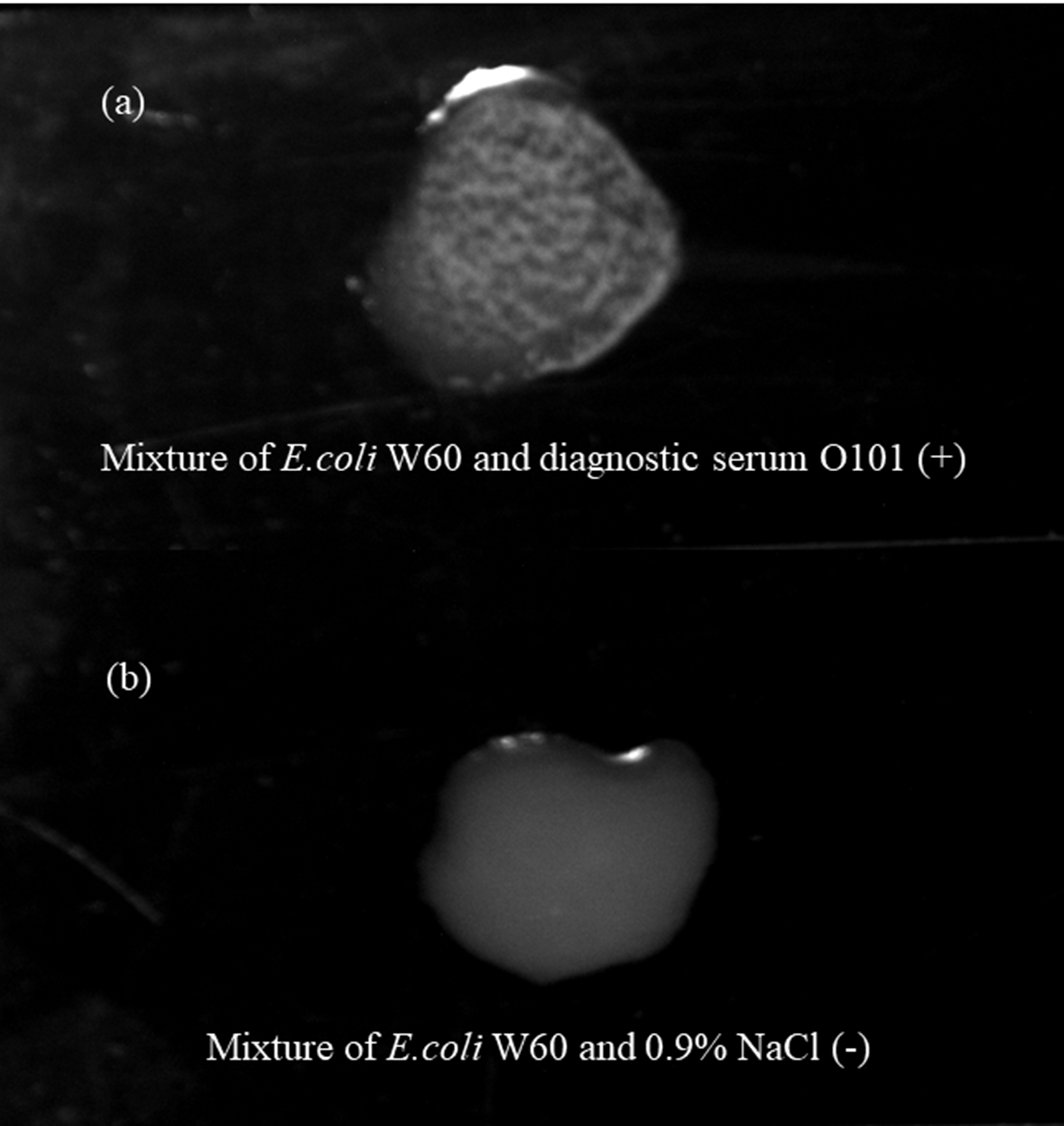

Supplement: Supplementary file 3 [file Image_2.TIF]

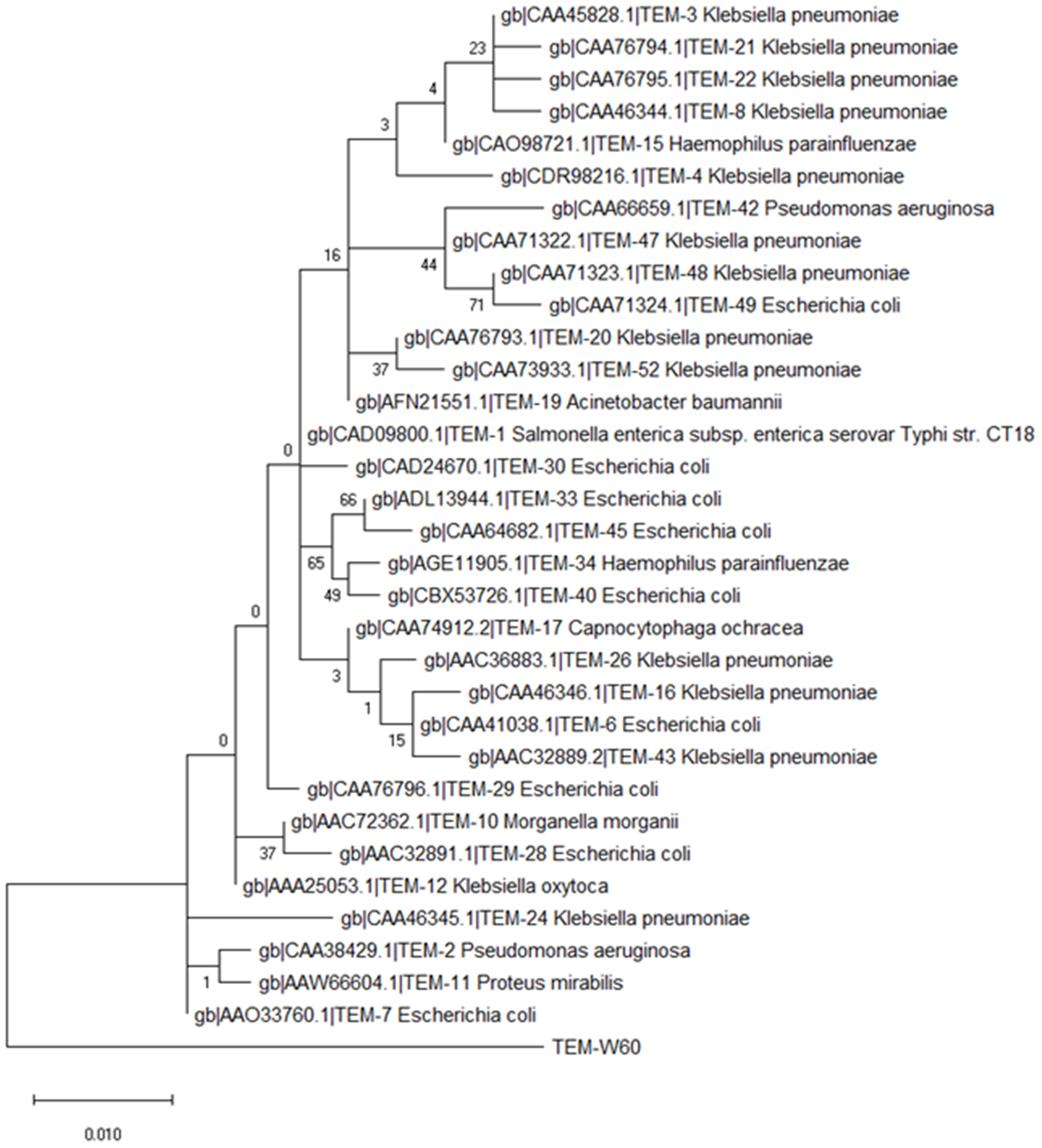

Supplement: Supplementary file 4 [file Image_3.TIF]

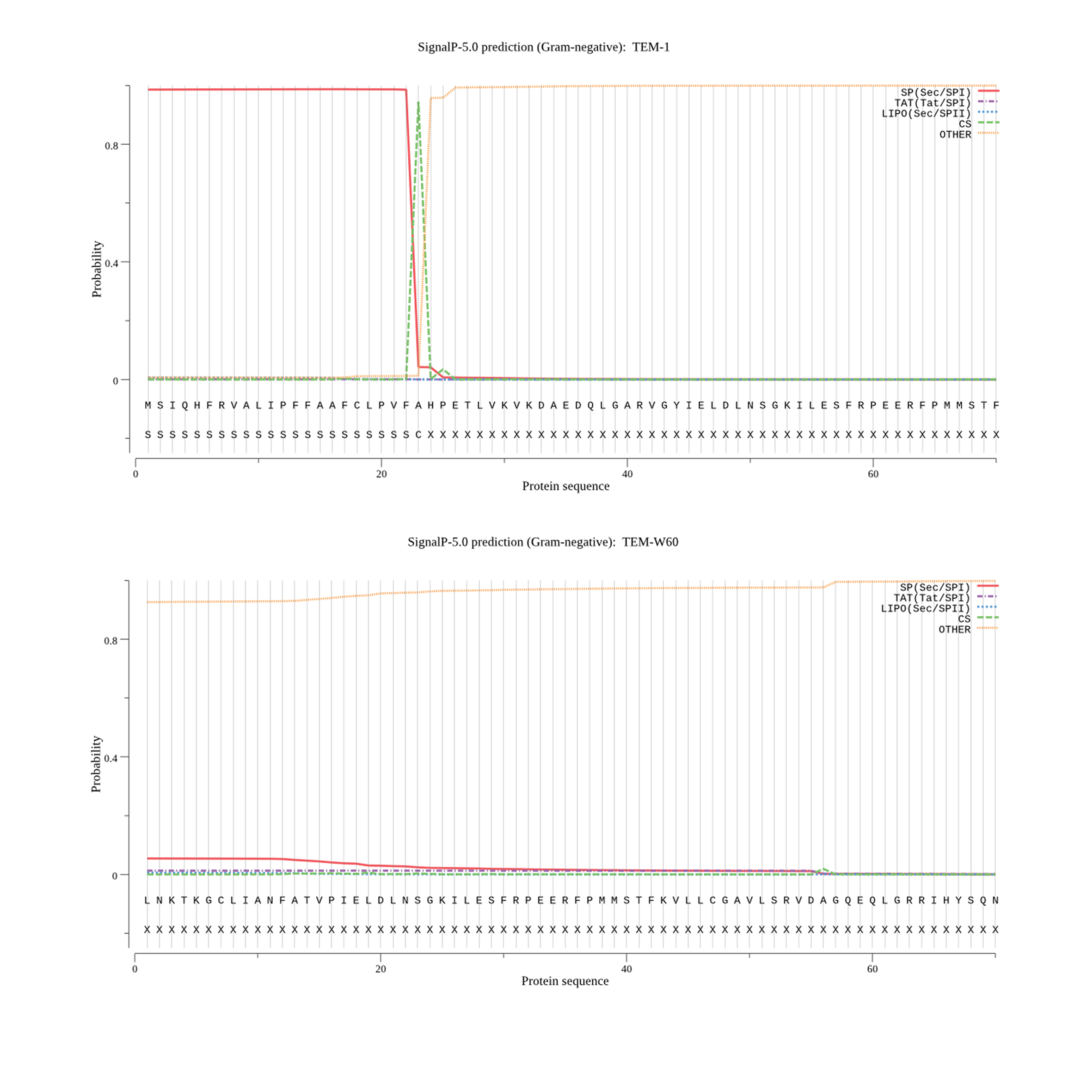

Supplement: Supplementary file 5 [file Image_4.TIF]
